# L-Carnitine Production Through Biosensor-Guided Construction of the *Neurospora crassa* Biosynthesis Pathway in *Escherichia coli*

**DOI:** 10.3389/fbioe.2021.671321

**Published:** 2021-04-16

**Authors:** Pierre Kugler, Marika Trumm, Marcel Frese, Volker F. Wendisch

**Affiliations:** ^1^Genetics of Prokaryotes, Faculty of Biology and CeBiTec, Bielefeld University, Bielefeld, Germany; ^2^Department of Chemistry, Organic and Bioorganic Chemistry (OCIII), Bielefeld University, Bielefeld, Germany

**Keywords:** carnitine, biosynthesis, biosensor, metabolic engineering, biosynthetic cascade, trimethyllysine

## Abstract

L-Carnitine is a bioactive compound derived from L-lysine and *S*-adenosyl-L-methionine, which is closely associated with the transport of long-chain fatty acids in the intermediary metabolism of eukaryotes and sought after in the pharmaceutical, food, and feed industries. The L-carnitine biosynthesis pathway has not been observed in prokaryotes, and the use of eukaryotic microorganisms as natural L-carnitine producers lacks economic viability due to complex cultivation and low titers. While biotransformation processes based on petrochemical achiral precursors have been described for bacterial hosts, fermentative *de novo* synthesis has not been established although it holds the potential for a sustainable and economical one-pot process using renewable feedstocks. This study describes the metabolic engineering of *Escherichia coli* for L-carnitine production. L-carnitine biosynthesis enzymes from the fungus *Neurospora crassa* that were functionally active in *E. coli* were identified and applied individually or in cascades to assemble and optimize a four-step L-carnitine biosynthesis pathway in this host. Pathway performance was monitored by a transcription factor-based L-carnitine biosensor. The engineered *E. coli* strain produced L-carnitine from supplemented L-*N*^ε^-trimethyllysine in a whole cell biotransformation, resulting in 15.9 μM carnitine found in the supernatant. Notably, this strain also produced 1.7 μM L-carnitine *de novo* from glycerol and ammonium as carbon and nitrogen sources through endogenous *N*^ε^-trimethyllysine. This work provides a proof of concept for the *de novo* L-carnitine production in *E. coli*, which does not depend on petrochemical synthesis of achiral precursors, but makes use of renewable feedstocks instead. To the best of our knowledge, this is the first description of L-carnitine *de novo* synthesis using an engineered bacterium.

## Introduction

L-Carnitine [(*R*)-3-hydroxy-4-trimethylaminobutyrate] is an essential compound in the intermediary metabolism of eukaryotes, which is involved in the transport of activated long-chain fatty acids and products of peroxisomal β-oxidation into the mitochondria for subsequent completion of β-oxidation (Vaz and Wanders, 2002). Only the L-isomer is physiologically functional in fatty acid transport (Reuter and Evans, 2012), while the other is linked to inhibitory or other toxic effects in different organisms (Rebouche, 1977, 1983; Gross et al., 1986; Li J.M. et al., 2019). In humans, 75% of the L-carnitine requirement is covered by the diet, while the remaining 25% are synthesized endogenously (Longo et al., 2016). Carnitine deficiency can lead to a wide range of symptoms that weaken the body (Magoulas and El-Hattab, 2012; Longo et al., 2016). Carnitine can be used as dietary supplement for the treatment of this deficiency (Flanagan et al., 2010; El-Hattab and Scaglia, 2015), but also as feed additive to improve livestock performance (Rehman et al., 2017; Ringseis et al., 2018a,b; Li L. et al., 2019), and as L-carnitine or acyl-L-carnitine esters in further pharmaceutical applications (DiNicolantonio et al., 2013; Chen et al., 2014; Song et al., 2017; Parvanova et al., 2018; Veronese et al., 2018). It is also marketed as food additive and dietary supplement for improving athletic performance and weight management as positive effects on physical performance and weight loss under conditions with disorders have been observed (Fielding et al., 2018), while an improvement in healthy individuals or athletes is debated (Gnoni et al., 2020). The carnitine market of US$ ∼170 million in 2018 is expected to grow by 4.8% annually (Grand View Research, 2019; The Insight Partners, 2020).

Racemic D,L-carnitine can be produced chemically from cheap epichlorohydrin and trimethylamine (Kabat et al., 1997), but to obtain enantiomerically pure L-carnitine, either chiral resolution or stereospecific synthesis is required. These approaches are not environmentally friendly, due to the number of reactions, and the need to use chiral starting materials or chiral auxiliaries (Meyer and Robins, 2005; Bernal et al., 2007). Achiral precursors such as crotonobetaine (dehydrated D,L-carnitine), γ-butyrobetaine, and 3-dehydrocarnitine are converted in highly regio- and enantioselective biotransformations under mild conditions (Bernal et al., 2016). These processes use *Pseudomonas* sp., *Proteus* sp., or *Escherichia coli* (Naidu et al., 2000) and are characterized by a significant reduction in environmental impact, e.g., 89% less waste to be incinerated, 82% less waste water, and 76% less salts (Meyer and Robins, 2005). Fermentative processes hold the potential for *de novo* biosynthesis from renewable feedstocks and to abandon the petrochemical synthesis of precursors. The use of eukaryotic microorganisms as natural L-carnitine producers suffers from low titers and demanding cultivation conditions with complex media (Wargo and Meadows, 2015; Bernal et al., 2016). Metabolic engineering of established bacterial producers such as *E. coli* holds the potential to establish *de novo* L-carnitine production in fermentative one-pot processes.

**FIGURE 1:**
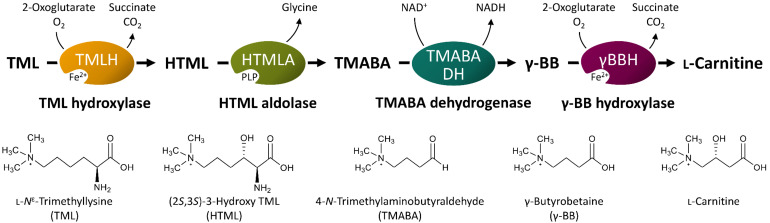
Schematic illustration of L-carnitine synthesis from the precursor TML. PLP, pyridoxal 5′-phosphate; NAD^+^, nicotinamide adenine dinucleotide (oxidized form); NADH, nicotinamide adenine dinucleotide (reduced form). Other abbreviated substances are written in full in the bottom of the figure. Modified from Strijbis et al. (2010).

Biosynthesis of L-carnitine in the filamentous fungus *Neurospora crassa* initiates with L-*N*^ε^-trimethyllysine (TML) (Horne and Broquist, 1973; Kaufman and Broquist, 1977; Vaz and Wanders, 2002). In eukaryotes, TML is the product of lysosomal or proteasomal degradation of proteins such as calmodulin, myosin, actin, cytochrome c, and histones, which contain *N*-methylated lysine residues from post-translational modification (Strijbis et al., 2010). TML is converted to L-carnitine in four enzymatic steps (Figure 1), starting with the hydroxylation in β-position by the TML hydroxylase (TMLH; EC 1.14.11.8) to yield (2*S*,3*S*)-3-hydroxy-TML (HTML) (Sachan and Hoppel, 1980; Vaz et al., 2001; Al Temimi et al., 2016; Reddy et al., 2017). In the next step, HTML is cleaved into glycine and 4-trimethylaminobutyraldehyde (TMABA) by HTML aldolase (HTMLA; EC 4.1.2. “X”). Instead of an aldolase with strict HTML specificity, serine hydroxymethyltransferases (SHMT) (Hulse et al., 1978; Henderson et al., 1982) and threonine aldolases (Strijbis et al., 2009; Franken et al., 2015) show side activities as HTMLA. In the third step, the NAD^+^-dependent TMABA dehydrogenase (TMABADH; EC 1.2.1.47) oxidizes TMABA to γ-butyrobetaine (γ-BB) as shown for *Pseudomonas* sp. 13CM (Hassan et al., 2008; Bari et al., 2013). Finally, stereoselective hydroxylation of γ-BB by γ-BB hydroxylase (γ-BBH; EC 1.14.11.1) yields L-carnitine. γ-BBH was not only found in mammals (Lindstedt, 1967; Lindstedt and Lindstedt, 1970), but also in the bacterium *Pseudomonas* sp. AK 1 (Lindstedt et al., 1967, 1970), where it is involved in utilization of γ-BB as the sole source of carbon and nitrogen for growth.

Transfer of the L-carnitine biosynthetic pathway from *N. crassa* to *Saccharomyces cerevisiae* suffered from low conversion of TML to L-carnitine (Franken et al., 2015). In a similar approach using different genes, production of L-carnitine from L-lysine in an *E. coli* whole cell biotransformation was described in a patent application (Kang et al., 2013). Discrepancies between these studies suggest that the *N. crassa* genes coding for γ-BBH gene and TMABADH have not been unequivocally identified (Kang et al., 2013; Franken et al., 2015), while genes encoding enzymes with TMLH and HTMLA activities are known.

In this study, the enzymes of the L-carnitine biosynthetic pathway from *N. crassa* and their corresponding genes were identified and functionally expressed in *E. coli*. This organism was chosen as production host because its natural interactions with L-carnitine are already well understood: It uses L-carnitine as compatible solute under hyperosmotic stress conditions and as terminal electron acceptor in anaerobic respiration yielding γ-BB (Seim et al., 1980; Wargo and Meadows, 2015). Uptake of L-carnitine is mediated by ATP-dependent ABC transport system ProU and to a lesser extent by ProP, a major facilitator superfamily (MFS) proton-metabolite symporter accepting proline, glycine betaine, stachydrine, pipecolic acid, ectoine, taurine, and L-carnitine (Verheul et al., 1998). Anaerobic respiration of L-carnitine involves the enzymes encoded in the divergent operons *caiTABCDE* and *fixABCX* (Jung and Kleber, 1991; Eichler et al., 1994; Preusser et al., 1999; Elssner et al., 2001; Jung et al., 2002; Bernal et al., 2007; Bracher et al., 2019; Kugler et al., 2020) that have been used in biotransformation processes of crotonobetaine or D-carnitine to L-carnitine (Castellar et al., 1998, 2001; Obón et al., 1999; Sevilla et al., 2005a,b; Bernal et al., 2007; Arense et al., 2013). We have recently developed a genetically encoded biosensor that responds in a dose-dependent manner to external L-carnitine in a concentration range of 0.1–100 μM by expression of the fluorescent reporter mVenus (Kugler et al., 2020). Its use to screen and score enzymes of L-carnitine biosynthesis in *E. coli* has been exemplarily shown for betaine:CoA ligases (Kugler et al., 2020). Here, this biosensor was applied to establish the L-carnitine biosynthetic pathway in *E. coli*. Besides one-pot biotransformation of TML to L-carnitine, L-carnitine could be produced without TML addition. To the best of our knowledge, this is the first fermentative *de novo* process for L-carnitine production.

## Materials and Methods

### Bacterial Strains, Recombinant DNA Work, Media, and Growth Conditions

A complete list of the bacterial strains and plasmids is given in Supplementary Table 2. *E. coli* DH5α was used as a cloning host, for plasmid propagation, and as the expression host for the TMABADH enzyme assay and SDS-PAGE. For cloning purposes, cells were grown in lysogeny broth (LB) medium (10 g L^–1^ tryptone, 5 g L^–1^ yeast extract, and 10 g L^–1^ sodium chloride) at 37°C on a shaking incubator (shaking frequency: 180 min^–1^, eccentricity: 25 mm). All media were supplemented with antibiotics where appropriate for selection: kanamycin (50 μg mL^–1^), tetracycline (10 μg mL^–1^), and chloramphenicol (25 μg mL^–1^).

For each tested enzyme of the carnitine biosynthetic pathway from *N. crassa*, a synthetic gene was designed based on the amino acid sequence. The codon usage in these genes was optimized based on codon usage tables^1^ (Nakamura, 2000) in a codon harmonization tool (Haupka, 2020). The DNA material of these artificial genes was ordered as gene synthesis from BioCat GmbH (Heidelberg, Germany). Artificial optimized 5′ untranslated regions (5′ UTRs) including the ribosome binding sites (RBS) were generated for each gene and vector combination with the online RBS calculator of the Salis Laboratory of Penn State University^2^ and were introduced *via* the oligonucleotides during cloning.

The standard molecular genetic techniques were performed as described in detail elsewhere (Green et al., 2012). Genes were amplified by PCR using the respective primers given in Supplementary Table 3. The oligonucleotides were supplied by Metabion GmbH (Planegg/Steinkirchen, Germany), and the PCR kit containing ALLin HiFi DNA Polymerase was purchased from highQu GmbH (Kraichtal, Germany) and used according to the manufacturer’s instructions. Plasmids were digested using restriction enzymes supplied by Thermo Fisher Scientific Inc. (Waltham, MA, United States) which were used according to the manufacturer’s protocols. Insertion of the amplified PCR products was done with Gibson assembly (Gibson et al., 2009). New vectors were confirmed by insert sequencing. The cloning of the vectors is described in detail in the Supplementary Material.

### Biosensor Experiments

The biosensor experiments were conducted as described earlier (Kugler et al., 2020). In short, M9 minimal medium with 24 g L^–1^ glycerol as the carbon source and supplemented antibiotics as stated above and 0.1 mM Isopropyl-β-D-thiogalactopyranosid (IPTG) was used for the cultivation at 37°C in 48-well microtiter FlowerPlates with the Biolector cultivation system (m2p-laboratories GmbH, Baesweiler, Germany) (Funke et al., 2009; Kensy et al., 2009). The medium was inoculated to an optical density at 600 nm (OD_600_) of 0.1 from precultures that were made in the M9 medium supplemented with 1% (v/v) LB medium. The formation of biomass was recorded as well as the fluorescence of the yellow fluorescent protein mVenus NB. The fluorescence signal was divided by the biomass signal to obtain the normalized fluorescence signal. The maximum normalized fluorescence signal observed during the whole cultivation was used for the evaluation. Unless stated otherwise, the maximum normalized fluorescence was detected between the middle and end of the exponential growth phase of the culture and for the controls (i.e., empty vector or supplemented water) in the stationary phase.

### Assay of TMABA Dehydrogenase Activity in *E. coli* Crude Extracts

*Escherichia coli* DH5α was used as host for the expression of the potential TMABA dehydrogenases. The enzymes were expressed from the pECXT99A-derived vectors containing the genes TMABADH.1S, TMABADH.1, or TMABADH.2. The cultivation was performed in 500 mL baffled shake flasks with 50 mL LB medium supplemented with antibiotics as stated above on a shaking incubator (37°C, shaking frequency: 180 min^–1^, eccentricity: 25 mm) with a starting OD_600_ of 0.1. At an OD_600_ of 0.6–0.7, expression was induced with 1 mM IPTG and the culture was continued up to an OD_600_ of ∼3.0 where the cells were harvested at the end of the exponential growth phase by centrifugation (10 min, 3,220 × *g*, 4°C). The supernatant was discarded, and the cell pellets were stored at −80°C until further use.

Frozen cell pellets were thawed on ice, resuspended in 1.8 mL lysis buffer [50 mM potassium phosphate buffer, 1 mM dithiothreitol (DTT); pH 7.5], and sonicated for 3 min on ice water (UP200S, Hielscher Ultrasonics GmbH, Teltow, Germany; amplitude 60%, cycle 0.5 s). The cell debris was removed by centrifugation (45 min, 28,000 × *g*, 4°C), and the extracts were stored on ice until use in the enzyme assay and for determination of protein concentration with Bradford Reagent (Sigma-Aldrich, St. Louis, MO, United States), using bovine serum albumin as the standard.

TMABA dehydrogenase activity was determined as described before (Bari et al., 2013) with slight modifications. The reaction was followed by measuring the formation of 1 mol NADH per consumed mol of TMABA photometrically at 340 nm (ε_*NADH*_ = 6,220 M^–1^ cm^–1^, d = 1 cm, 30°C). The reaction mixture of 1 mL consisted of 150 mM glycine-NaOH buffer (pH 9.5), 2 mM NAD^+^, 0.4 mM TMABA iodide, and 250 μL crude extract. The basal absorption change was followed for 3 min and then the reaction was initialized by the addition of TMABA. Where necessary, the crude extract was diluted with the lysis buffer to achieve a rate of absorption change during the measurement in the range of 0.03–0.15 per min.

### Protein Gel Analysis

The crude extracts from the TMABA dehydrogenase activity assay were further analyzed by sodium dodecyl sulfate polyacrylamide gel electrophoresis (SDS-PAGE) as described elsewhere (Green et al., 2012) using the Mini-PROTEAN Tetra Cell #1658000EDU System from Bio-Rad Laboratories Inc. (Hercules, CA, United States). The amount of protein used per sample was 12 μg, and the PageRuler Prestained Protein Ladder 10–180 kDa (Thermo Fisher Scientific Inc., Waltham, MA, United States) was used as molecular weight marker.

### Carnitine Production Experiment With Recombinant *E. coli* Expressing the Carnitine Biosynthetic Pathway From *N. crassa*

The cultivation was done in 100 mL baffled shake flasks with 10 mL modified M9 medium with 30 g L^–1^ glycerol as carbon source and with antibiotics as described above. Nitrogen is the limiting factor for biomass formation in the composition of the M9 medium, which is why NH_4_Cl was supplemented in this experiment to double the concentration to 2 g L^–1^ to reach a higher biomass in this cultivation. The cultures were inoculated to an OD_600_ of 0.1 from precultures that were made in standard M9 medium supplemented with 1% (v/v) LB medium and placed in a shaking incubator (37°C, 180 min^–1^ shaking frequency, 25 mm eccentricity). At an OD_600_ of 0.5–0.6, expression was induced with 0.1 mM IPTG and the substrate TML was added at a concentration of 1 mM. Cultivation was continued for 48 h, and then a 2 mL sample was drawn from which the cell pellet and supernatant were obtained by centrifugation (10 min, 12,000 × *g*, 4°C). After washing the cells with saline (9 g L^–1^ NaCl), the cells were pelleted again by centrifugation (10 min, 12,000 × *g*, 4°C) and then all samples were frozen at −20°C until analysis *via* LC-MS.

### LC-MS Measurement

The supernatant samples were thawed on ice and then centrifuged (10 min, 12,000 × *g*, 4°C) to separate possible precipitates from freezing. Subsequently, the undiluted samples were analyzed by LC-MS. The cell pellets were resuspended in 800 μL of ice-cold 80% (v/v) methanol, mixed with 0.35 g of 0.2 mm zirconia-silica beads (BioSpec Products Inc., Bartlesville, OK, United States) in 1.5 mL microtubes, and cooled on ice. The suspensions were placed in the Digital Disruptor Genie Cell Disruptor (Scientific Industries Inc., Bohemia, NY, United States), and the cells were disrupted for 3 min at full speed. Next, cell debris and beads were separated by centrifugation (10 min, 12,000 × *g*, 4°C). The methanol was evaporated from 600 μL of the resulting supernatant in a vacuum concentrator (Eppendorf AG, Hamburg, Germany) for 4 h at 45°C. The residue was re-dissolved in 120 μL water and analyzed by LC-MS. For the LC-MS measurement, an Agilent 6220 TOF-MS with a Dual ESI-source and a 1200 HPLC system (Agilent Technologies, Inc., Santa Clara, CA, United States) with a Hypersil Gold C18 column (1.9 μm, 50 × 2.1 mm; Thermo Fisher Scientific Inc., Waltham, MA, United States) was used. Solvent A consisted of 94.9% (v/v) water, 5% (v/v) acetonitrile, and 0.1% (v/v) formic acid, and solvent B was composed of 5% (v/v) water, 94.9% (v/v) acetonitrile, and 0.1% (v/v) formic acid. A gradient was started at 0% B when 5 μL sample was injected and increased to 98% B over 11 min, went back to 0% B in 0.5 min, which was held for 3.5 min to a total run time of 15 min. The flow rate was 300 μL min^–1^, and column oven temperature was at 40°C. The extended dynamic range mode was used with a Dual-ESI source, operating with a spray voltage of 2.5 kV. The data were analyzed with MassHunter Workstation Software Version B.07.00 (Agilent Technologies, Inc., Santa Clara, CA, United States). Carnitine was identified in an accurate mass measurement from its mass to charge ratio (*m/z*) of 162.11267 (deviation <5 ppm to calculated *m/z* of 162.11247). The mass spectrum of L-carnitine is shown in Supplementary Figure 2. The quantitative analysis was performed using a set of L-carnitine standard samples with the concentrations (in μM): 0, 1.56, 3.13 6.25, 12.5, 25, and 50. From the resulting total ion count (TIC), a regression line was calculated (*R*^2^ = 0.998), which was used as the calibration curve. Intracellular carnitine concentrations were calculated from the carnitine concentration determined for the cell pellet extract and the total cell volume of the cell pellet, which was calculated from the final OD_600_ of the 2 mL samples at 48 h fermentation and the OD specific total cell volume for starved *E. coli* cells in the stationary phase of 3.3 μL mL^–1^ OD_600_^–1^ (Volkmer and Heinemann, 2011).

### Analytical Quantification of Glycerol in the Carnitine Production Experiment

Samples were drawn at 24 h and 48 h cultivation time. The supernatant was collected by centrifugation (10 min, 12,000 × *g*, 4°C) and frozen at −20°C until analysis *via* HPLC. For analysis, the samples were thawed on ice and then centrifuged (10 min, 12,000 × *g*, 4°C) to separate possible precipitates from freezing. Subsequently, the undiluted samples were analyzed on an HPLC system (1200 series, Agilent Technologies Deutschland GmbH, Böblingen, Germany) with an organic acid resin column (300 mm × 8 mm) and the corresponding pre-column (40 mm × 8 mm) (Chromatographie-Service GmbH, Langerwehe, Germany) heated to 60°C. The mobile phase was 5 mM sulfuric acid in water (Milli-Q grade) with an isocratic flow rate of 0.8 mL min^–1^. A refractive index detector was used for molecule detection. A calibration curve (*R*^2^ = 0.999) was generated from glycerol standards with the concentrations (in g L^–1^): 1, 2, 4, 8, 16, and 32.

### Reagents

Unless specified, all chemical standard reagents were purchased in the highest grade available from Sigma-Aldrich (St. Louis, MO, United States) and Carl Roth GmbH + Co., KG (Karlsruhe, Germany). L-*N*^ε^ -trimethyllysine was purchased from Glentham Life Sciences Ltd. (Corsham, United Kingdom), and L-carnitine and γ-butyrobetaine were ordered at Sigma-Aldrich (St. Louis, MO, United States). The TMABA iodide was prepared from 4-aminobutyraldehyde diethyl acetal (Acros Organics B.V.B.A, Fair Lawn, NJ, United States) as described in the Supplementary Material.

## Results

### Selection and Cloning of the L-Carnitine Biosynthetic Pathway Genes From *Neurospora crassa*

The selection of the potential enzymes for the L-carnitine biosynthetic pathway in this study was based on two sets of *N. crassa* genes that were previously considered for a transfer of the pathway to heterologous hosts (Kang et al., 2013; Franken et al., 2015). An overview of the enzymes and their respective genes used in this study is given for each step in Supplementary Table 1. Both sets share the enzymes for the first two steps (TMLH and HTMLA). The TMLH has been previously cloned and confirmed in *S. cerevisiae* (Swiegers et al., 2002), and for the HTMLA, the gene of the *N. crassa* SHMT was selected, as it additionally possesses aldolase activity (Kruschwitz et al., 1993; Franken et al., 2015). In case of the TMABADH, a third variant (TMABADH.1) was tested here in addition to the versions TMABADH.1S (Kang et al., 2013) and TMABADH.2 (Franken et al., 2015). Both TMABADH.1S and TMABADH.1 are based on the ORF NCU00378; however, TMABADH.1 (549 AA; RefSeq: XP_957264.2) is longer than TMABADH.1S (495 AA; XP_957264.1), which results from a reannotation extending the ORF to a start codon 153 bp upstream. For γ-BBH, two different enzymes termed γBBH.1 (Kang et al., 2013) and γBBH.2 (Franken et al., 2015) were selected from the literature and chosen for this study.

A codon-harmonized gene and corresponding optimized 5′ UTRs including the RBS were designed for each variant using a codon harmonization tool (Haupka, 2020) and codon usage tables^3^ (Nakamura, 2000), and the online RBS calculator of the Salis Laboratory of Penn State University (see text footnote 1).

### Screening for Functional γ-Butyrobetaine Hydroxylases

**FIGURE 2:**
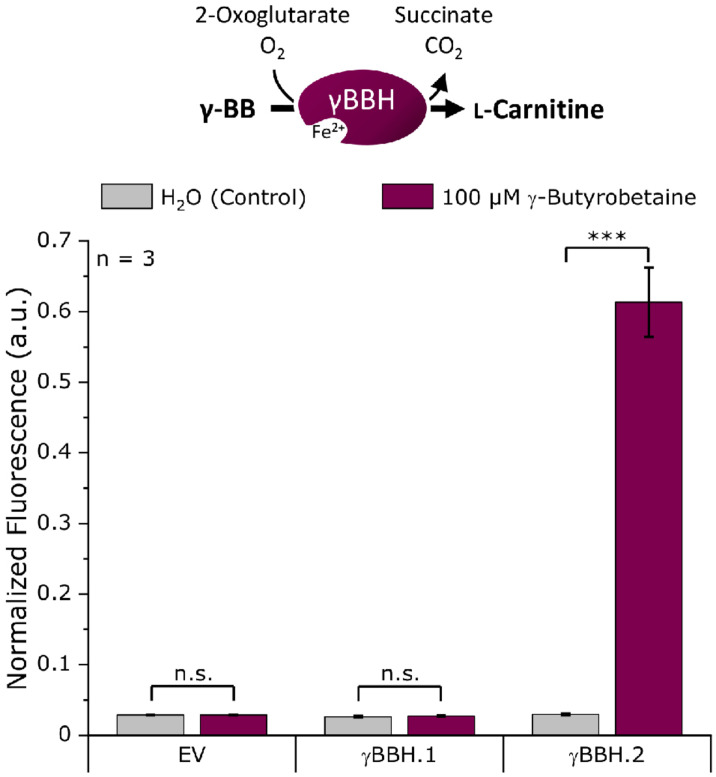
Biosensor screening of potential γ-butyrobetaine hydroxylases from *N. crassa*. The artificial genes γBBH.1 and γBBH.2 derived from the ORFs NCU02196 and NCU12046, respectively, were cloned into pECXT99A and tested in the L-carnitine biosensor strain *E. coli* Δ*cai-fix* (pGP2-Sensor1-*caiCD*). The empty vector (EV) of pECXT99A was used as a control. The maximum normalized fluorescence signal observed during the cultivation process with 100 μM γ-butyrobetaine or water as the control is shown. Error bars indicate the standard deviation of the mean of three replicates. Three asterisks (^∗∗∗^) indicate a *p*-value ≤ 0.001 in a student’s *t*-test; n.s., not significant.

For the screening of the γ-butyrobetaine hydroxylases, the genes γBBH.1 and γBBH.2 (Supplementary Table 1) were cloned into the expression plasmid pECXT99A. To score γ-butyrobetaine hydroxylase activity, an L-carnitine biosensor strain was used, and fluorescence was measured upon extracellular addition of γ-BB. Specifically, the enzymes were tested for L-carnitine formation from γ-BB in the L-carnitine biosensor strain *E. coli* BW25113 Δ*cai-fix* (pGP2-Sensor1-*caiCD*) (see Supplementary Table 2), which was transformed with pECXT99A-γBBH.1, pECXT99A-γBBH.2, or pECXT99A as empty vector control. A microbioreactor cultivation was performed using the Biolector system (m2p-laboratories GmbH, Baesweiler, Germany) (Funke et al., 2009; Kensy et al., 2009) with the resulting strains in M9 minimal medium supplemented with 100 μM γ-BB or water as control and the maximum normalized mVenus fluorescence was measured (Figure 2). M9 minimal medium was selected for all cultivation experiments because it significantly increases the utility and sensitivity of the biosensor used (Kugler et al., 2020).

As expected, no L-carnitine biosensor fluorescence signal was measured in the empty vector control by the addition of γ-BB. While γBBH.1 showed no activity when γ-BB was supplemented, an L-carnitine biosensor fluorescence signal could be detected for γBBH.2 upon addition of γ-BB. Thus, γBBH.2 was confirmed in this screening as functionally active γ-butyrobetaine hydroxylase and used for further work on the L-carnitine biosynthetic pathway.

### Screening for Functional TMABA Dehydrogenases

**FIGURE 3:**
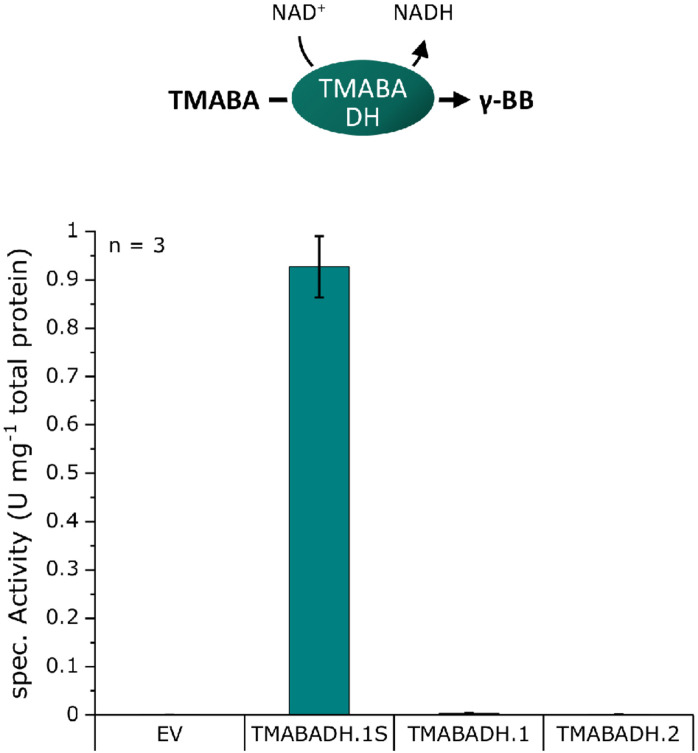
TMABADH activities in crude extracts of *E. coli* DH5α transformed with pECXT99A derivatives expressing potential TMABADH from *N. crassa*. TMABADH.1S and TMABADH.1 were derived from NCU00378, and TMABADH.2 was derived from NCU03415. pECXT99A was used as the empty vector control (EV). Means of three replicates are shown. Error bars were calculated by error analysis from the standard deviations of the enzyme assays and Bradford measurements.

An *in vitro* photometric enzyme assay based on cell extracts was chosen for screening TMABA dehydrogenase activity, as it is easily feasible due to the co-conversion of NAD^+^ to NADH in the TMABADH reaction (Figure 3). A screening with the biosensor as for the γ-BBH was not chosen because external supplementation of aldehydes can have toxic effects on the cells due to their high reactivity and it is uncertain that the aldehyde is taken up by the cells (Kunjapur and Prather, 2015). *E. coli* DH5α was transformed with pECXT99A-based plasmids harboring the dehydrogenases TMABADH.1S, TMABDH.1, or TMABADH.2 (Supplementary Table 1) and with pECXT99A as the empty vector control. Cell extracts were prepared from the induced strains and examined in an enzyme assay in which the reaction was photometrically monitored by measuring the formation of NADH (Figure 3).

As expected, no activity could be detected in the empty vector control, and neither TMABADH.1 nor TMABADH.2 showed activity. By contrast, TMABA dehydrogenase activity was revealed for TMABADH.1S (Figure 3). Since TMABADH.1 and TMABADH.1S share a great similarity on the amino acid level, it was speculated whether the expression of TMABADH.1 was affected by the N-terminal modification compared to TMABADH.1S. To investigate this, an SDS-PAGE was performed from the same cell extracts. Indeed, protein abundance of TMABADH.1 was significantly lower as compared to TMABADH.1S and TMABADH.2 (Supplementary Figure 1). Since TMABADH.1S could be expressed well and showed about 0.9 U mg^–1^ activity, it was selected for further work on the carnitine biosynthetic pathway.

### Assembly of the L-Carnitine Biosynthetic Pathway

**FIGURE 4:**
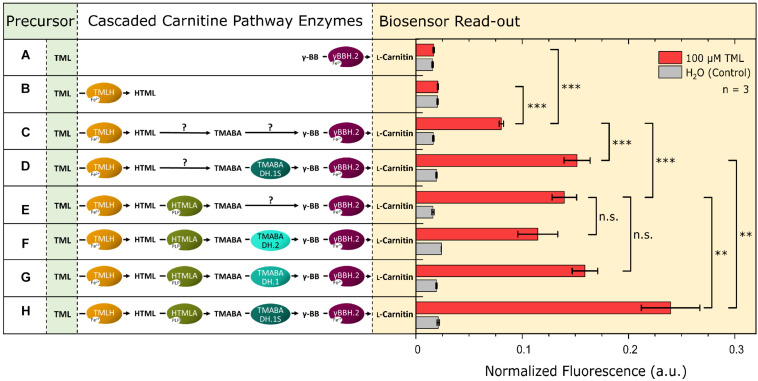
Assembly of the four steps of the L-carnitine biosynthetic pathway of *N. crassa* in *E. coli*. The expression of a gene encoding a cascaded enzyme is depicted by an oval on the left side. Arrows marked with a “?” indicate that the reaction may proceed *via* endogenous enzymes although no dedicated gene was overexpressed. Plasmid pPLib3 was used for the individual expression of TMLH (pPLib3-TMLH; Combinations B, C, and D) and together with HTMLA as synthetic operon (pPLib3-TMLH-HTMLA; Combinations E, F, G, and H) and as empty vector control (pPLib3; Combination A). pECXT99A was used to express γBBH.2 individually (pECXT99A-γBBH.2; Combinations A, C, and E) or in a synthetic operon with one of the TMABADH variants (pPLib3-TMABADH.1S-γBBH.2; Combinations D and H, pPLib3-TMABADH.1-γBBH.2; Combination G, or pPLib3-TMABADH.2-γBBH.2; Combination F) and as empty vector control (Combination B). The shown enzyme combinations were tested in the L-carnitine biosensor strain *E. coli* Δ*cai-fix* (pGP2-Sensor1-*caiCD*). The maximum normalized fluorescence signal observed during the cultivation process with 100 μM TML (red columns) or water (gray columns) as the control is shown. Error bars indicate the standard deviation of the mean of three replicates. Three asterisks (^∗∗∗^) indicate a *p*-value ≤ 0.001 in a student’s *t*-test, ^∗∗^*p*-value ≤ 0.01, and n.s., not significant.

After efficient enzymes for the last and penultimate step of the L-carnitine biosynthetic pathway had been identified, enzymes for the first two steps were selected: TML hydroxylase and an HTML aldolase (Supplementary Table 1). With the biosensor as a read-out tool for L-carnitine formation, the pathway was gradually assembled in the biosensor strain and the formation of L-carnitine from externally supplemented TML was studied. Additionally, this experimental setup was used to investigate the TMABADH variants *in vivo* in combination with the other pathway enzymes. The L-carnitine biosensor strain *E. coli* BW25113 Δ*cai-fix* (pGP2-Sensor1-*caiCD*) was transformed with different plasmid combinations to assemble the full pathway. The TMABADH variants were included to validate the results from the *in vitro* assay in an *in vivo* biolector cultivation experiment. M9 minimal medium supplemented with 100 μM TML or water as control was used and the maximum normalized mVenus fluorescence was measured (Figure 4). Individual expression of γBBH.2 or TMLH (combinations A and B, respectively) did not result in an L-carnitine biosensor signal upon addition of TML as precursor. Cascading of all four enzymes *in vivo* gave a strong L-carnitine biosensor signal (combination H) indicating that the pathway operated as anticipated.

Surprisingly, cascades with TMLH, but lacking one or more (active) enzymes (combination C, D, E, F, and G) also showed L-carnitine biosensor signal output, albeit it was significantly lower than that of the complete cascade (combination H). This suggests that enzymes present in the *E. coli* host strain are active in the conversion of HTML to γ-BB. However, currently their identities remain unknown. For further work on the pathway, the cascade TML hydroxylase/HTML aldolase/TMABADH.1S/γBBH.2 (combination H) was chosen.

### Improving the L-Carnitine Biosynthesis Pathway for *de novo* Production by *E. coli*

**FIGURE 5:**
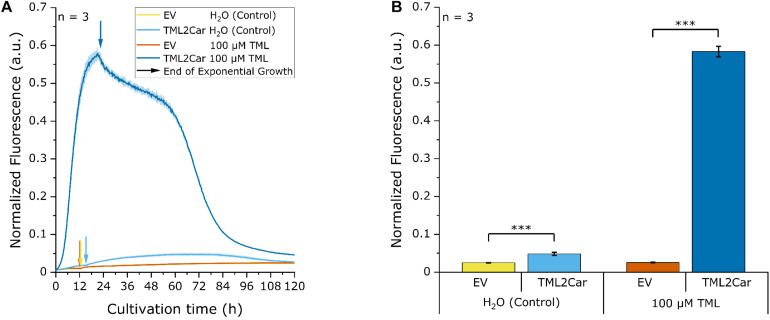
Biosensor experiment with the synthetic L-carnitine biosynthesis pathway operon. The pathway genes were cloned in the order of the respective reaction steps into pECXT99A resulting in plasmid pECXT99A-TML2Car and tested in the L-carnitine biosensor strain *E. coli* Δ*cai-fix* (pGP2-Sensor1-*caiCD*). The empty vector (EV) of pTrc99A was used as a control. In the cultivation, either 100 μM TML was supplemented, or water was used as control. **(A)** Development of the normalized fluorescence signal over the cultivation process. The yellow (EV, H_2_O) and orange lines (EV, 100 μM TML) are largely overlapping. The end of the exponential growth phase and transition to the stationary growth phase is marked with arrows for each condition. **(B)** Analogous to the previous readouts, the maximum normalized fluorescence signal for each cultivation was extracted from **(A)** and analyzed for significance of values between TML2Car and the respective EV control. Error bars indicate the standard deviation of the mean of three replicates. Three asterisks (^∗∗∗^) indicate a *p*-value ≤ 0.001 in a student’s *t*-test.

After having selected the enzymes for the L-carnitine biosynthetic pathway, expression of the four genes was optimized by combining the individual genes in a single plasmid as a synthetic operon. The plasmid pTrc99A was used as backbone and the previously identified genes for the pathway (TMLH, HTMLA, TMABADH.1S, and γBBH.2; Figure 4, combination H) were cloned with optimized 5′UTRs in a single synthetic operon. The gene order was the same as that of the reaction steps, and the constructed plasmid for conversion of TML to L-carnitine was called pTrc99A-TML2Car. The plasmid was transformed in the L-carnitine biosensor strain *E. coli* BW25113 Δ*cai-fix* (pGP2-Sensor1-*caiCD*) and tested in parallel with pTrc99A as empty vector control in a biolector experiment. The medium was supplemented with either 100 μM TML or water as control, and the normalized mVenus fluorescence was monitored over the cultivation time (Figure 5A). In the strain harboring the L-carnitine biosynthetic pathway (labeled TML2Car), the supplementation of 100 μM TML resulted in a fluorescence response of the L-carnitine biosensor. The maximum signal (Figure 5B) was more than twofold higher than the signal previously measured for the pathway when its genes were expressed from two separate plasmids (Figure 4, Combination H). Interestingly, strain TML2Car showed a significantly increased biosensor signal compared to the empty vector strain even when no TML was supplemented (Figure 5B, H_2_O control). This may indicate *de novo* L-carnitine formation from endogenous TML. In contrast to the measurement with supplemented TML, the maximum normalized fluorescence was detected in the stationary phase and not at the end of the exponential growth phase (Figure 5A). The origin of TML in the *E. coli* host is unknown. While interesting from a physiological point of view, it was not required for the L-carnitine biosynthetic cascade developed here.

### LC-MS Analysis of L-Carnitine Production by Recombinant *E. coli* Expressing the L-Carnitine Biosynthetic Pathway from *N. crassa*

**FIGURE 6:**
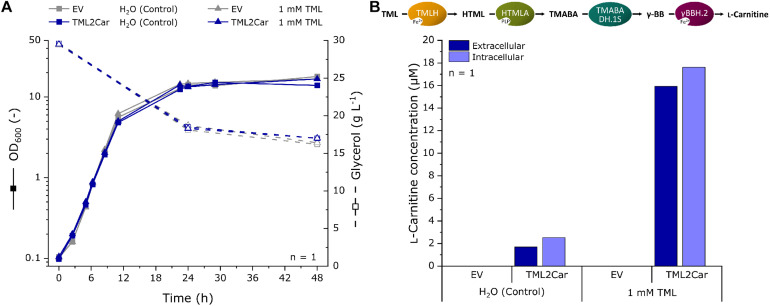
Fermentation for L-carnitine production in modified M9 minimal medium with supplementation of 1 mM TML or water as a control using recombinant *E. coli* expressing the L-carnitine biosynthetic pathway from *N. crassa.* The pathway was expressed in *E. coli* BW25113 Δ*cai-fix* from plasmid pTrc99A-TML2Car. The empty vector (EV) pTrc99A was used as control. **(A)** Growth curve and residual glycerol concentrations of the fermentation experiment. **(B)** LC-MS analysis of the extracellular and intracellular L-carnitine concentration at the end of the cultivation after 48 h. L-carnitine was identified by its mass to charge ratio (*m/z*) of 162.11247. The detection limit was 0.5 μM. Intracellular concentrations were determined from the L-carnitine concentration in cell pellet extracts and the calculated total cell volume of the pellet.

The heterologous expression of the genes for the fullL-carnitine biosynthesis cascade by the plasmid pTrc99A-TML2Car revealed L-carnitine production not only from the supplemented precursor TML, but also without it, providing evidence of *de novo* L-carnitine biosynthesis (Figure 5). To further substantiate these results, L-carnitine formation was studied in a shake flask production experiment, and extracellular and intracellular L-carnitine concentrations were analyzed by LC-MS. The strains were similar to those used in the previous experiment but lacked the biosensor plasmid. *E. coli* strains BW25113 Δ*cai-fix* (pTrc99A-TML2Car) and empty vector control BW25113 Δ*cai-fix* (pTrc99A) were cultivated for 48 h in modified M9 minimal medium with increased levels of glycerol and ammonium as carbon and nitrogen sources and with and without supplementation of 1 mM TML. LB or another complex medium was not used to keep the cultivation comparable to the biosensor experiments and to avoid potential TML and carnitine sources in the medium, e.g., yeast extract. The growth and carbon source consumption were followed over the cultivation time (Figure 6A). The EV strain grew slightly faster with a maximum growth rate of 0.52 h^–1^ and an average final OD_600_ of 17.3, whereas the TML2Car strain grew at a rate of 0.46 h^–1^ to an average OD_600_ of 15.3 at the end of the fermentation after 48 h, with all strains converting ∼50% of the provided carbon source glycerol.

The supernatant and cells pellets were analyzed by LC-MS, and the extracellular and intracellular L-carnitine concentrations were determined (Figure 6B). The mass of L-carnitine (*m/z* ratio 162.11247) could be scored in the supernatants and cells of the strain expressing the L-carnitine biosynthetic pathway (labeled TML2Car), but not in the empty vector control strain. An extracellular concentration of 1.7 μM was produced without supplementation of TML. When 1 mM of the precursor was supplemented, 15.9 μM L-carnitine could be found in the supernatant (1.6% molar yield as judged by the L-carnitine and TML concentrations in the culture medium). The intracellular L-carnitine concentrations were similar to the supernatants but slightly higher.

## Discussion

Implementing a cascade of enzymes from *N. crassa* into *E. coli* enabled biotransformation of TML to L-carnitine as demonstrated using a genetically encoded L-carnitine biosensor and by LC-MS analysis. Moreover, the metabolically engineered *E. coli* strain enabled *de novo* production of L-carnitine from a mineral salts medium with glycerol and ammonium as carbon and nitrogen sources.

The ability of *E. coli* to synthesize sufficient TML for *de novo* production of L-carnitine came as a surprise since *E. coli* synthesizes TML to a much lesser extent than eukaryotic cells (Klagsbrun and Furano, 1975; Zhang et al., 2018). Methyltransferase PrmA methylates two lysyl residues of protein L11 from the large ribosomal subunit (Colson et al., 1979; Vanet et al., 1994) and free TML accumulates upon degradation of methylated protein L11 similar to TML release in eukaryotes (Vaz and Wanders, 2002). Our observation of accumulation of L-carnitine in the stationary phase is consistent with release of TML by protein degradation, which primarily occurs in this growth phase. Alternatively, it is possible that PrmA methylates free lysine to free TML as a side reaction as demonstrated for the RuBisCo large subunit *N*-methyltransferase (RBCMT) from *Pisum sativum* (Trievel et al., 2003).

Endogenous enzymes of *E. coli* complemented the four-step pathway when only TMLH and γ-BBH were provided (Figure 4; Combination C), thus, enzymes with (side) activities as HTML aldolase and TMABA dehydrogenase exist in *E. coli*. The low-specificity L-threonine aldolase LtaE, which cleaves several L-3-hydroxy-α-amino acids, like HTML, to glycine and a corresponding aldehyde and which may accommodate molecules larger than L-threonine in its large substrate binding pocket (di Salvo et al., 2014), may exhibit side activity as HTML aldolase. Alternatively, this activity may be catalyzed by *E. coli*’s SHMT GlyA, which shares 48% amino acid identity with the *N. crassa* SHMT that was used in this study (Schirch et al., 1985). In the case of TMABA dehydrogenase, numerous aldehyde dehydrogenases native to *E. coli* may possess this side activity (Sophos and Vasiliou, 2003). The most likely candidates among them may be γ-aminobutyraldehyde dehydrogenase PatD that is active with unmethylated TMABA (γ-aminobutyraldehyde) or with betaine aldehyde (Gruez et al., 2004; Samsonova et al., 2005) and PuuC, a non-specific aldehyde dehydrogenase that oxidizes all aldehydes in putrescine catabolism including γ-aminobutyraldehyde (Schneider and Reitzer, 2012). It remains to be shown if overexpression of the genes for these enzymes is beneficial for *de novo* production of L-carnitine since accumulation of intermediates in the conversion of TML to L-carnitine was not observed.

The *E. coli* strain constructed here produced 1.7 μM L-carnitine *de novo*, which is fourfold higher than by biotransformation from 100 μM supplemented TML by recombinant *S. cerevisiae* (Franken et al., 2015). However, our finding that addition of 1 mM TML to the *E. coli* strain constructed here improved L-carnitine production about 10-fold (1.7 μM as compared to around 16 μM) suggested that the supply of the precursor TML may limit *de novo* production. It remains to be shown if overexpression of endogenous *prmA* or heterologous expression of the RBCMT gene from *P. sativum* increases *de novo* production of L-carnitine by the *E. coli* strain constructed here. Alternatively, a *bona fide* L-lysine methyltransferase (K-NMT) yielding TML may be used. Such an enzyme has been reported for *N. crassa* (Borum and Broquist, 1977). The gene NCU03826 that was speculated to encode K-NMT in a patent application (Kang et al., 2013) did not result in a biosensor response that would indicate improved L-carnitine levels and the supplementation of lysine and methionine as precursors did not change this outcome (data not shown). Consistent with this finding, NCU03826 did not function as K-NMT in the *S. cerevisiae* strain (Franken et al., 2015). Thus, the gene for a *bona fide* K-NMT remains elusive.

Metabolic engineering to improve provision of the precursor metabolites L-lysine and *S*-adenosyL-methionine (SAM) may increase *de novo* production of L-carnitine by the *E. coli* strain constructed here. Strategies to overproduce L-lysine by *E. coli* are well established (Wendisch, 2020) and an *E. coli* strain overproducing L-lysine to a titer of 194 g L^–1^ from glucose and ammonium has recently been described (Ye et al., 2020). Regeneration of SAM, the major co-substrate for methyltransferases, using the renewable feedstock methanol as source of the methyl group proved very efficient in *E. coli* (Okano et al., 2020). Enzyme engineering of *E. coli* SAM synthetase to reduce product inhibition and increase catalytic activity is a complementary strategy (Wang et al., 2019).

The L-carnitine biosensor fluorescence signal suggested high conversion of L-carnitine from intracellular TML (Figure 5B) as values were comparable to direct L-carnitine supplementation (Kugler et al., 2020). However, only low L-carnitine concentrations were found intra- and extracellularly (Figure 6B), suggesting that the biosensor is sensitive to low intracellular L-carnitine levels and that the export of L-carnitine out of the *E. coli* cell may also limit biotransformation and its *de novo* production. Consistent with this hypothesis, transport limitations have also been observed for biotransformation processes converting achiral precursors such as crotonobetaine to L-carnitine using recombinant *E. coli* strains (Bernal et al., 2007). For biotransformations, this can be overcome by cell permeabilization, for example, with polyethylenimine (Cánovas et al., 2005; Bernal et al., 2007). However, strategies for production of L-carnitine *de novo* have to maintain the cell’s integrity. Deletion of the gene for Braun’s lipoprotein from the outer membrane of *E. coli* improved L-carnitine production from crotonobetaine without affecting cell growth and metabolism (Bernal et al., 2007; Ni et al., 2007). To prevent re-uptake of L-carnitine secreted from the *E. coli* cell, the genes coding for L-carnitine uptake systems ProU and ProP were deleted (Verheul et al., 1998; Sevilla et al., 2005a). Engineering L-carnitine export was possible for biotransformation, but is not suitable for *de novo* production. This is due to the fact that CaiT is an antiporter catalyzing exchange of intracellular and extracellular trimethylammonium compounds such as L-carnitine, crotonobetaine, and γ-butyrobetaine, while it does not catalyze uniport of L-carnitine out of the *E. coli* cell (Jung et al., 2002). A transport system for export of L-carnitine operating in uniport mode is currently not known.

In this work, a proof of concept for the *de novo* L-carnitine production in *E. coli* was shown. The developed fermentation does not depend on petrochemical synthesis of achiral precursors, but instead makes use of renewable feedstocks. Implementation of the L-carnitine biosynthesis pathway was guided by the recently developed L-carnitine biosensor, which allowed the identification of the enzymes and their assembly into an optimized biosynthetic pathway in the form of an enzyme cascade. The biosensor additionally made it possible to identify the precursor supply and product export as current bottlenecks that need to be addressed to further advance L-carnitine *de novo* production by *E. coli*. To the best of our knowledge, this is the first description of L-carnitine *de novo* synthesis using an engineered bacterium.

## Data Availability Statement

The original contributions presented in the study are included in the article/Supplementary Material, further inquiries can be directed to the corresponding author/s.

## Author Contributions

PK designed the study. PK, MT, and MF conducted the experiments. VW provided funding. MF and VW provided resources. PK, MF, and VW drafted the manuscript. PK and VW finalized the manuscript. All authors read and approved the final version of the manuscript.

## Conflict of Interest

The authors declare that the research was conducted in the absence of any commercial or financial relationships that could be construed as a potential conflict of interest.

## Footnotes

1 www.kazusa.or.jp/codon/

2 https://salislab.net/software/

3 www.kazusa.or.jp/codon/
