# Supplementary material for: L-Carnitine Production Through Biosensor-Guided Construction of the Neurospora crassa Biosynthesis Pathway in Escherichia coli
Source: Front Bioeng Biotechnol. 2021 Apr 16;9:671321. doi: 10.3389/fbioe.2021.671321 (PMC8085414; doi:10.3389/fbioe.2021.671321)
Supplement: Supplementary file 1 [file Data_Sheet_1.PDF]

Supplementary Material

L-Carnitine production through biosensor-guided construction of the *Neurospora crassa* biosynthesis pathway in *Escherichia coli*

Pierre Kugler[1], Marika Trumm[1], Marcel Frese[2] and Volker F. Wendisch[1*]

[1]Genetics of Prokaryotes, Faculty of Biology & CeBiTec, Bielefeld University, 33615 Bielefeld, Germany

[2]Organic and Bioorganic Chemistry (OCIII), Department of Chemistry, Bielefeld University, 33615 Bielefeld, Germany

*** Correspondence:**
Volker F. Wendisch
volker.wendisch@uni-bielefeld.de

1 Selection of the Carnitine Biosynthetic Pathway Genes from *N. crassa*

Supplementary Table S1. Overview of the potential genes for each step of the carnitine biosynthetic pathway from *N. crassa* that were tested for functionality in *E. coli* in this study.

Step	EC number	Name	Gene name (ORF)	Protein RefSeq ID	Reference
1	1.14.11.8	TMLH	NCU03802	XP_961191.3	(Kang et al., 2013; Franken et al., 2015; Swiegers et al., 2002)
2	4.1.2.‘X’	HTMLA	NCU02274	XP_959918.1	(Kang et al., 2013; Franken et al., 2015)
3	1.2.1.47	TMABADH.1S	NCU00378	XP_957264.1	(Kang et al., 2013)
		TMABADH.1	NCU00378	XP_957264.2	Sequence update of XP_957264.1
		TMABADH.2	NCU03415	XP_956862.1	(Franken et al., 2015)
4	1.14.11.1	γBBH.1	NCU02196	XP_959590.1	(Kang et al., 2013)
		γBBH.2	NCU12046	XP_011395335.1	(Franken et al., 2015)

2 Protein Gel Analysis (SDS-PAGE)

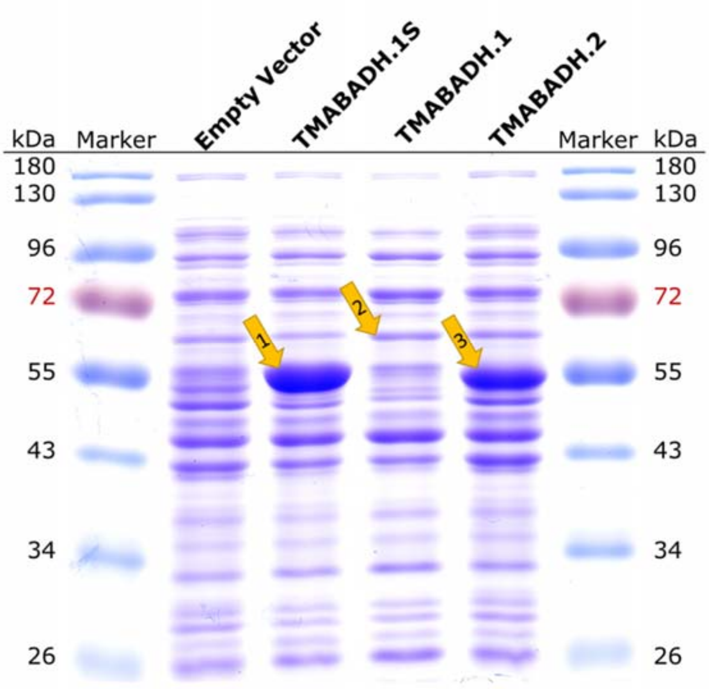

Supplementary Figure S1. Protein gel analysis of the three different TMABADHs in *E. coli* DH5α crude extracts from the TMABADH activity assay. The amount of protein used per sample was 12 µg. Expected masses of additional protein bands: TMABADH.1S: 53.8 kDa (Arrow 1), TMABADH.1: 59.8 kDa (Arrow 2), TMABADH.2: 54.0 kDa (Arrow 3). Marker: PageRuler Prestained Protein Ladder 10-180 kDa (Thermo Fisher Scientific Inc., Waltham, MA, USA). *E. coli* DH5α was used as host for the expression from pECXT99A derived expression vectors containing the genes TMABADH.1S, TMABADH.1 or TMABADH.2. The plasmid pECXT99A was used as empty vector control.

3 LC-MS Analysis of Carnitine Production

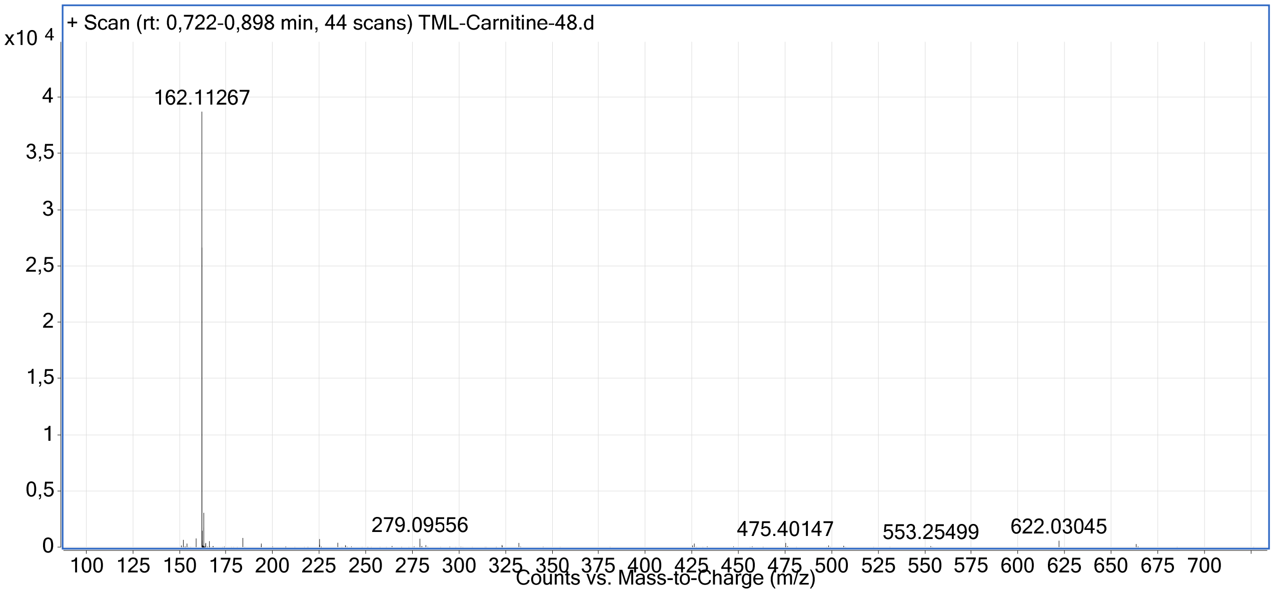

Supplementary Figure S2. Mass spectrum of L-carnitine in the accurate mass measurement. An Agilent 6220 TOF-MS with a Dual ESI-source and a 1200 HPLC system (Agilent Technologies, Inc., Santa Clara, CA, USA) with a Hypersil Gold C18 column (1.9 µm, 50 × 2.1 mm; Thermo Fisher Scientific Inc., Waltham, MA, USA) was used in positive mode. Based on the retention time and the measured ion mass of 162.11267 (deviation <5 ppm (1.23 ppm) from calculated ion mass 162.11247), the identity of the molecule as L-carnitine was confirmed.

4 Cloning and Vector construction

A complete list of the bacterial strains and plasmids is given in the Supplementary Table S2. Genes were amplified by PCR using the respective primers given in the Supplementary Table S3. Insertion of the amplified PCR products was done with Gibson assembly (Gibson et al., 2009).

Supplementary Table S2. Strains and plasmids used in this study.

Strains and plasmids	Relevant characteristics	Reference
**Strains**		
***E. coli* DH5α**	F-, *Δ(argF-lac)169*, φ80d*lacZ58*(M15), *ΔphoA8*, *glnX44*(AS), λ^-, *deoR481*, *rfbC1*?, *gyrA96*(NalR), *recA1*, *endA1*, *thiE1*, *hsdR17*	(Hanahan, 1983)
***E. coli* BW25113**	(wild type) F-, *Δ(araD-araB)567*, *ΔlacZ4787*(::rrnB-3), λ^-, *rph-1*, *Δ(rhaD-rhaB)568*, *hsdR514*	CGSC (Keio)(Datsenko and Wanner, 2000; Baba et al., 2006)
E. coli* BW25113 Δ*cai-fix	BW25113 Δ*cai-fix*. The carnitine biotransformation pathway is deleted in this strain	(Kugler et al., 2020)
**Plasmids**		
**pGP2-Sensor1-*caiCD***	Carnitine biosensor plasmid<br><br>Km^R, p15A *ori*, constitutive expression of *caiF* and *caiCD* by P_{hom} of *Corynebacterium glutamicum, mVenus NB* under control of P_{fix}.	(Kugler et al., 2020)
**pPLib2-*sfGFP***	Expression vector<br><br>Cm^R, P_{lac}, *sfGFP*, ColDF13 *ori*	(Kugler et al., 2020)
**pPLib3**	Expression vector.<br><br>Cm^R, P_{trc}, *lacI*, ColDF13 *ori*	This study

**pPLib3-TMLH**	pPLib3 derivative for expression of a synthetic TMLH gene derived from *N. crassa* ORF NCU03802 with an optimized RBS	This study
**pPLib3-HTMLA**	pPLib3 derivative for expression of a synthetic HTMLA gene derived from *N. crassa* ORF NCU02274 with an optimized RBS	This study
**pPLib3-TMLH-HTMLA**	pPLib3-TMLH derivative extended for expression of a synthetic HTMLA gene derived from *N. crassa* ORF NCU02274 with an optimized RBS in an artificial operon	This study
**pECXT99A**	Expression vector<br>Tet^R, P_{trc}, *lacI*, ColE1 *ori*	(Kirchner and Tauch, 2003)
**pECXT99A-TMABADH.1S**	pECXT99A derivative for expression of the synthetic TMABADH.1S gene derived from *N. crassa* ORF NCU00378 and protein sequence XP_957264.1 (short version) with an optimized RBS	This study
**pECXT99A-TMABADH.1**	pECXT99A derivative for expression of the synthetic TMABADH.1 gene derived from *N. crassa* ORF NCU00378 and protein sequence XP_957264.2 with an optimized RBS	This study
**pECXT99A-TMABADH.2**	pECXT99A derivative for expression of the synthetic TMABADH.2 gene derived from *N. crassa* ORF NCU03415 with an optimized RBS	This study
**pECXT99A-γBBH.1**	pECXT99A derivative for expression of the synthetic γBBH.1 gene derived from *N. crassa* ORF NCU02196 with an optimized RBS	This study
**pECXT99A-γBBH.2**	pECXT99A derivative for expression of the synthetic γBBH.2 gene derived from *N. crassa* ORF NCU12046 with an optimized RBS	This study
**pECXT99A-TMABADH.1S-γBBH.2**	pECXT99A derivative for expression of the synthetic TMABADH.1S gene derived from *N. crassa* ORF NCU00378 and protein sequence XP_957264.1 (short version) with an optimized RBS in an artificial operon with the synthetic γBBH.2 gene derived from *N. crassa* ORF NCU12046 with an optimized RBS	This study

**pECXT99A-TMABADH.1-γBBH.2**	pECXT99A derivative for expression of the synthetic TMABADH.1 gene derived from *N. crassa* ORF NCU00378 and protein sequence XP_957264.2 with an optimized RBS in an artificial operon with the synthetic γBBH.2 gene derived from *N. crassa* ORF NCU12046 with an optimized RBS	This study
**pECXT99A-TMABADH.2-γBBH.2**	pECXT99A derivative for expression of the synthetic TMABADH.2 gene derived from *N. crassa* ORF NCU03415 with an optimized RBS in an artificial operon with the synthetic γBBH.2 gene derived from *N. crassa* ORF NCU12046 with an optimized RBS	This study
**pTrc99A**	Expression vector<br><br>Amp^R, P_{trc}, *lacI*, ColE1 *ori*	(Amann et al., 1988)
**pTrc99A-TML2Car**	pTrc99A derivative for expression of an artificial operon of TMLH, HTMLA, TMABADH.1S and γBBH.2 genes derived from *N. crassa* ORF NCU03802, NCU2274, NCU00378 and NCCU12046, respectively, each with an optimized RBS	This study

Supplementary Table S3. Oligonucleotides used in this study.

Oligonucleotide	Application	Sequence (5’ → 3’)
**pPLib3_BB_F1_fw**	pPLib3 Fragment 1 from pPLib2-*sfGFP*	ATATGTATCCGCTCATGCTCCTTCGacggcactcctcagccaag
**pPLib3_BB_F1_rv**	pPLib3 Fragment 1 from pPLib2-*sfGFP*	gcactgaaatctagagcggttcagtag
**pPLib3_BB_F2_fw**	pGP2 Fragment 2 from pECXT99A	actgaaccgctctagatttcagtgcGACACCATCGAATGGTGCAAAACC
**pPLib3_BB_F2_rv**	pGP2 Fragment 2 from pECXT99A	CGAAGGAGCATGAGCGGATAC
**TMLH_pPLib3_fw**	Cloning of pPLib3-TMLH / pPLib3-TMLH-HTMLA / pTrc99A-TML2Car	atggaattcgagctcggtacccgggTCTGACAGTTTTTAAGAGATATTTAAGGAGGTTTTTTatgcgccctcaggttgttggtgctattttgaggtcccg
**TMLH_pPLib3_rv**	Cloning of pPLib3-TMLH	GCATGCCTGCAGGTCGACTCTAGAGttagccggtaacgcgtggcag
**TMLH_HTMLA_rv**	Cloning of pPLib3-TMLH-HTMLA	TTATTTAATAAACCGGCTAGCAGAttagccggtaacgcgtggc
**HTMLA_pPLib3_fw**	Cloning of pPLib3-HTMLA	ATTCGAGCTCGGTACCCGGGCCCGCTCACTCAACAACAATACACTATAAGGAGGTTTTTTatgagcacctactccctgtc
**HTMLA_pPLib3_rv**	Cloning of pPLib3-HTMLA	GCATGCCTGCAGGTCGACTCTAGAGttacagacctgcgtcgtatcgccag
**HTMLA_TMLH_fw**	Cloning of pPLib3-TMLH-HTMLA	ggctaaTCTGCTAGCCGGTTTATTAAATAAGGAGGTTTTTTatgagcacctactccctgtccg
**TMABADH.1S_pECXT99A_fw**	Cloning of pECXT99A-TMABADH.1S /	TCGAGCTCGGTACCCGGGCGCTCACAGCAGAAACGAGGACAAATAAGAAGGAGGGATACGatggaagtggaactgaccgc

	pECXT99A-TMABADH.1S-γBBH.2	
**TMABADH.1/S_pECXT99A_rv**	Cloning of pECXT99A-TMABADH.1S / pECXT99A-TMABADH.1	GCATGCCTGCAGGTCGACTCTAGAGTTATGCTGCCAGGTTCACGTGGATGG
**TMABADH.1_pECXT99A_fw**	Cloning of pECXT99A-TMABADH.1	GGAATTCGAGCTCGGTACCCGGGTCTAAAACATTGTTAAAGGAGGTATTTTTATGAACCTGTTCCTGCGCCCATC
**TMABADH.2_pECXT99A_fw**	Cloning of pECXT99A-TMABADH.2	GAGCTCGGTACCCGGGCTCGGAGTTCCAGGTTAAGAAATCTATAAGAAAGGAGCCATATTTATGTCCTCCAACGTGTTCG
**TMABADH.2_pECXT99A_rv**	Cloning of pECXT99A-TMABADH.2	GCCTGCAGGTCGACTCTAGAGTTAGTTCAGCTTGATTGCCACGG
**yBBH.1_pECXT99A_fw**	Cloning of pECXT99A-γBBH.1	ATGGAATTCGAGCTCGGTACCCGGGATTAAGGGGAGGATTTTATTAAGAAGGAGGTAACAAatggcaaccgcagcagtgc
**yBBH.1_pECXT99A_rv**	Cloning of pECXT99A-γBBH.1	GCATGCCTGCAGGTCGACTCTAGAGttagtagccgccgccgccc
**yBBH.2_pECXT99A_fw**	Cloning of pECXT99A-γBBH.2	GAATTCGAGCTCGGTACCCGGGGTACTATTAACACACAACTAAAACAAAGGAGGTCTTTTatgcttcgccagcttggagc
**yBBH.2_pECXT99A_rv**	Cloning of pECXT99A-γBBH.2 / pECXT99A-TMABADH.1S-γBBH.2 / pECXT99A-TMABADH.1-γBBH.2 / pECXT99A-TMABADH.2-γBBH.2 / pTrc99A-TML2Car	GCATGCCTGCAGGTCGACTCTAGAGttacgcgttccagttaacagtgcc
**TMABADH.1S_yBBH.2_rv**	Cloning of pECXT99A-TMABADH.1S-γBBH.2	gAcgaagcatAAAAAAAACCTCCTTAATCTTTACTAATGTTAGAACCCAGCCAGttatgctgccaggttcacgtggatgg
**yBBH.2_TMABADH.1S_fw**	Cloning of pECXT99A-TMABADH.1S-γBBH.2	CTGGCTGGGTTCTAACATTAGTAAAGATTAAGGAGGTTTTTTTatgcttcgTcaActtggagcagcAccactggcgc

**yBBH.2_TMABADH.1_fw**	Cloning of pECXT99A-TMABADH.1-γBBH.2	TCCACGTGAACCTGGCAGCATAACTCTGGCTGGGTTCTAACATTAGTAAAG
**TMABADH.2_pECXT99A_fw**	Cloning of pECXT99A-TMABADH.2-γBBH.2	CGAGCTCGGTACCCGGGTCTCCGATAGGAAATTTTTAGAGAAGAGTAAGGAGGTATTTATTATGTCCTCCAACGTGTTCG
**TMABADH.2_yBBH.2_rv**	Cloning of pECXT99A-TMABADH.2-γBBH.2	TTAGTTCAGCTTGATTGCCACGG
**yBBH.2_TMABADH.2_fw**	Cloning of pECXT99A-TMABADH.2-γBBH.2	CCGTGGCAATCAAGCTGAACTAACTCTGGCTGGGTTCTAACATTAGTAAAG
**HTMLA_TML2Car_rv**	Cloning of pTrc99A-TML2Car	CCTTATATAAATCTACGCAAATCCGCAAttacagacctgcgtcgtatcgcc
**TMABADH.1S_TML2Car_fw**	Cloning of pTrc99A-TML2Car	gtaaTTGCGGATTTGCGTAGATTTATATAAGGAGGTACTTTTatggaagtggaactgaccgcacc

4.1 Construction of pPLib3 and pPLib3-Based Expression Vectors

Plasmid pPLib3 was constructed from two fragments which were assembled using the Gibson method. Fragment 1 contained the Cm^R and the CloDF13 *ori* amplified from pPLib2-*sfGFP* using the primers pPLib3_BB_F1_fw and pPLib3_BB_F1_rv. The second fragment consisted of *lacI*, P_{trc}, the multiple cloning site and the *rrnB* terminators 1 and 2 and was amplified from pECXT99A with the primers pPLib3_BB_F2_fw and pPLib3_BB_F2_rv.

To construct the pPLib3-based expression plasmids, the vector was digested with BamHI. pPLib3-TMLH was cloned using primers TMLH_pPLib3_fw and TMLH_pPLib3_rv. The HTMLA gene was amplified for pPLib3-HTMLA by the primers HTMLA_pPLib3_fw and HTMLA_pPLib3_rv. The plasmid pPLib3-TMLH-HTMLA was assembled from two inserts. TMLH was amplified using the primers TMLH_pPLib3_fw and TMLH_HTMLA_rv and HTMLA was amplified with the primers HTMLA_TMLH_fw and HTMLA_pPLib3_rv.

4.2 Construction of pECXT99A-Based Expression Vectors

The vector pECXT99A was digested with BamHI. For the construction of pECXT99A-TMABADH.1S the synthetic gene TMABADH.1S was amplified with the primers TMABADH.1S_pECXT99A_fw and TMABADH.1S_pECXT99A_rv. The plasmid pECXT99A-

TMABADH.1 was generated after amplification of the synthetic TMABADH.1 gene with the primers TMABADH.1_pECXT99A_fw and TMABADH.1_pECXT99A_rv. TMABADH.2 was amplified for the cloning of pECXT99A-TMABADH.2 with the primers TMABADH.2_pECXT99A_fw and TMABADH.2_pECXT99A_rv.

γBBH.1 was amplified using the primers yBBH.1_pECXT99A_fw and yBBH.1_pECXT99A_rv to construct pECXT99A-γBBH.1. For the generation of pECXT99A-γBBH.2 the primers yBBH.2_pECXT99A_fw and yBBH.2_pECXT99A_rv were used to amplify the synthetic γBBH.2 gene.

pECXT99A-TMABADH.1S-γBBH.2 was constructed from two fragments, one containing the TMABADH.S1 gene and the other the γBBH.2 gene, which were amplified with the primers TMABADH.1S_pECXT99A_fw and TMABADH.1S_yBBH.2_rv and yBBH.2_TMABADH.1S_fw and yBBH.2_pECXT99A_rv, respectively.

The plasmid pECXT99A-TMABADH.1-γBBH.2 was constructed based on pECXT99A-TMABADH.1, which was digested with XbaI. γBBH.2 was then inserted after amplification with primers yBBH.2_TMABADH.1_fw and yBBH.2_pECXT99A_rv.

pECXT99A-TMABADH.2-γBBH.2 was generated from two inserts, one consisted of the TMABADH.2 gene and the other of the γBBH.2 gene, which were amplified with the primers TMABADH.2_pECXT99A_fw and TMABADH.2_yBBH.2 rv and yBBH.2_TMABADH.2_fw and yBBH.2_pECXT99A_rv, respectively.

4.3 Construction of pTrc99A-TML2Car

For the construction of pTrc99A-TML2Car the vector pTrc99A was digested with BamHI and two fragments were inserted. The first contained the genes TMLH and HTMLA and was amplified from pPLib3-TMLH-HTMLA with the primers TMLH_pPLib3_fw and HTMLA_TML2Car_rv. The second fragment was amplified from pECXT99A-TMABADH.1S-γBBH.2 with the primers TMABADH.1S_TML2Car_fw and yBBH.2_pECXT99A_rv and contained TMABADH.1S and γBBH.2.

5 Synthetic genes used in this study

>TMLH

atgcgccctcaggttgttggtgctattttgaggtcccgcgcggttgtaagccgccagccactgtcccgcaccca
catcttcgctgctgttaccgttgctaagtcctcctccccagctcagaactcccgccgcaccttctcctcctcct
tccgccgcctgtacgagccaaaggctgagatcaccgctgagggcctggagctgtccccaccacaggctgttacc
ggcggcaagcgcaccgttctgccaaacttctggctgcgcgacaactgccgctgcaccaagtgcgttaaccagga
caccctgcagcgcaacttcaacaccttcgctatcccatccgacatccacccaaccaaggttgaggctaccaagg
agaacgttaccgttcagtggtccgacaaccacacctccacctacccatggccattcctgtccttctacctgacc
tccaacgctcgcggccacgagaacgaccagatctccctgtggggctccgaggctggctcccgcccaccaaccgt
ttccttcccacgcgttatggcttccgaccagggcgttgctgacctgaccgctatgatcaaggagttcggcttct
gcttcgttaaggacaccccacacgacgacccagacgttacccgccagctgctggaacgtatcgcgttcatccgc
gttactcactacggcggcttctacgacttcaccccagacctggctatggctgacaccgcttacaccaacctggc
tctgccagctcacaccgacaccacctacttcaccgacccagctggcctgcaggctttccacctgctggagcaca
aggctgctccatcccgcccaccaccaccaccaccaccaccaccaccaccatccgaggagaaggaggctgctggc
tccgctgctggcgaggctgctgctgctgctgagggcggcaagtccctgctggttgacggcttcaacgctgctcg
catcctgaaggaggaggacccacgcgcttacgagatcctgtcctccgttcgcctgccatggcacgcttccggca
acgagggcatcaccatcgctccagacaagctgtacccagttctggagctgaacgaggacaccggcgagctgcac
cgcgttcgctggaacaacgacgaccgcggcgttgttccattcggcgagaagtactccccatccgagtggtacga
ggctgctcgcaagtgggacggcatcctgcgccgcaagtcctccgagctgtgggttcagctggagccaggcaagc
cactgatcttcgacaactggcgcgttttacacggccgctccgcgttctccggcatccgtcgcatctgcggcggc
tacatcaaccgcgacgacttcatctcccgctggcgcaacaccaactacccacgctccgaggttctgccacgcgt
taccggctaa

>HTMLA

atgagcacctactccctgtccgaaactcacaaggcaatgctggaacattctcttgtggaatccgatccacaggt
ggcagaaatcatgaagaaggaagttcagcgccagcgcgaatccatcatcctgatcgcatccgaaaacgtgacct
ctcgagcagtgttcgacgcactgggctccccaatgtccaacaagtactctgaaggtcttccaggcgcacgctac
tatggtggcaaccagcacatcgatgaaatcgaagttctgtgccagaaccgagcacttgaagcattccacctgga
tccaaagcagtggggtgtgaatgttcagtgcctttccggctctcctgcaaacctgcaggtgtaccaggcaatca
tgccagtgcacggccgactgatgggtcttgatctgccacacggtggccatctttcccacggttaccagacccca
cagcgcaagatcagcgctgtgagcacctacttcgaaaccatgccataccgcgtgaacattgatactggtctgat
cgattacgacaccctggaaaagaacgcacagctgttccgcccaaaggtgctggtggcaggtaccagcgcatact
gccgactcattgattacgaacgcatgcgcaagattgcagattccgttggcgcttaccttgtggtggacatggct
cacatttccggcctgattgcatccgaagttatcccatctccattcctgtacgcagacgtggtgaccaccaccac
tcacaagagcctgcgaggccctcgaggcgcaatgatcttcttccgccgcggtgtgcgctccgttgatgcaaaga
ccggcaaggaaaccctgtacgatcttgaagataagatcaacttctccgtgttccctggtcaccagggtggccca
cacaaccacaccatcaccgcacttgcagttgcactgaagcaggctgcatccccagaattcaaggaataccagca
gaaggtggttgcaaacgcaaaggctctggaaaagaagctgaaggaactgggctacaagctggtgagcgatggca
ctgatagccacatggtgctggttgatcttcgcccaatcggcgtggacggtgcacgagttgaattcctgcttgaa
cagatcaacattacctgcaacaagaacgcagttccaggcgataagtctgcactgaccccaggcggtctgcgaat
tggtaccccagctatgacctcccgaggcttcggcgaagcagatttcgaaaaggtggcagtgttcgtggacgaag
ctgtgaagctgtgcaaggaaatccaggcttccctgccaaaggaagctaacaagcagaaggatttcaaggcaaag

atcgcaacctctgacattccacgcatcaacgaactgaagcaggaaattgcagcatggtctaacaccttcccact
gccagttgaaggctggcgatacgacgcaggtctgtaa

>TMABADH.1S

atggaagtggaactgaccgcaccaaacggcaagaagtggatgcagccactgggcctgttcatcaacaacgaatt
cgtgaagtccgcaaacgaacagaagctgatctccatcaacccaaccaccgaagaagaaatctgctccgtgtacg
cagcaaccgcagaagatgtggatgcagcagtgtccgcagcacgcaaggcattccgccacgaatcctggaagtcc
ctgtccggcaccgaacgcggcgcactgatgcgcaagctggcagatctggtggcagaaaacgcagaaatcctggc
aaccatcgaatgcctggataacggcaagccataccagaccgcactgaacgaaaacgtgccagaagtgatcaacg
tgctgcgctactacgcaggctacgcagataagaacttcggccaggtgatcgatgtgggcccagcaaagttcgca
tacaccgtgaaggaaccactgggcgtgtgcggccagatcatcccatggaactacccactggatatggcagcatg
gaagctgggcccagcactgtgctgcggcaacaccgtggtgctgaagctggcagaacagaccccactgtccgtgc
tgtacctggcaaagctgatcaaggaagcaggcttcccaccaggcgtgatcaacatcatcaacggccacggccgc
gaagcaggcgcagcactggtgcagcacccacaggtggataagatcgcattcaccggctccaccaccaccggcaa
ggaaatcatgaagatggcatcctacaccatgaagaacatcaccctggaaaccggcggcaagtccccactgatcg
tgttcgaagatgcagatctggaactggcagcaacctggtcccacatcggcatcatgtccaaccagggccagatc
tgcaccgcaacctcccgcatcctggtgcacgaaaagatctacgatgaattcgtggaaaagttcaaggcaaaggt
gcaggaagtgtccgtgctgggcgatccattcgaagaatccaccttccacggcccacaggtgaccaaggcacagt
acgaacgcgtgctgggctacatcaacgtgggcaaggaagaaggcgcaaccgtgatgatgggcggcgaaccagca
ccacagaacggcaagggcttcttcgtggcaccaaccgtgttcaccaacgtgaagccaaccatgaagatcttccg
cgaagaaatcttcggcccatgcgtggcaatcaccaccttcaagaccgaagaagaagcactgaccctggcaaacg
attccatgtacggcctgggcgcagcactgttcaccaaggatctgacccgcgcacaccgcgtggcacgcgaaatc
gaagcaggcatggtgtgggtgaactcctccaacgattccgatttccgcatcccattcggcggcgtgaagcagtc
cggcatcggccgcgaactgggcgaagcaggcctggcaccatactgcaacgtgaagtccatccacgtgaacctgg
cagcataa

>TMABADH.1

atgaacctgttcctgcgcccatcccgcccaggctccgtgccactgtggcgccacctgtacaagaccaagggcaa
gcaggtgtaccacgtgcagctgtccttacgcaccttctcctcctccccattcttcaccaacatcataaagc
caaacatggaagtggaactgaccgcaccaaacggcaagaagtggatgcagccactgggcctgttcatcaacaac
gaattcgtgaagtccgcaaacgaacagaagctgatctccatcaacccaaccaccgaagaagaaatctgctccgt
gtacgcagcaaccgcagaagatgtggatgcagcagtgtccgcagcacgcaaggcattccgccacgaatcctgga
agtccctgtccggcaccgaacgcggcgcactgatgcgcaagctggcagatctggtggcagaaaacgcagaaatc
ctggcaaccatcgaatgcctggataacggcaagccataccagaccgcactgaacgaaaacgtgccagaagtgat
caacgtgctgcgctactacgcaggctacgcagataagaacttcggccaggtgatcgatgtgggcccagcaaagt
tcgcatacaccgtgaaggaaccactgggcgtgtgcggccagatcatcccatggaactacccactggatatggca
gcatggaagctgggcccagcactgtgctgcggcaacaccgtggtgctgaagctggcagaacagaccccactgtc
cgtgctgtacctggcaaagctgatcaaggaagcaggcttcccaccaggcgtgatcaacatcatcaacggccacg
gccgcgaagcaggcgcagcactggtgcagcacccacaggtggataagatcgcattcaccggctccaccaccacc
ggcaaggaaatcatgaagatggcatcctacaccatgaagaacatcaccctggaaaccggcggcaagtccccact
gatcgtgttcgaagatgcagatctggaactggcagcaacctggtcccacatcggcatcatgtccaaccagggcc
agatctgcaccgcaacctcccgcatcctggtgcacgaaaagatctacgatgaattcgtggaaaagttcaaggca

aaggtgcaggaagtgtccgtgctgggcgatccattcgaagaatccaccttccacggcccacaggtgaccaaggc
acagtacgaacgcgtgctgggctacatcaacgtgggcaaggaagaaggcgcaaccgtgatgatgggcggcgaac
cagcaccacagaacggcaagggcttcttcgtggcaccaaccgtgttcaccaacgtgaagccaaccatgaagatc
ttccgcgaagaaatcttcggcccatgcgtggcaatcaccaccttcaagaccgaagaagaagcactgaccctggc
aaacgattccatgtacggcctgggcgcagcactgttcaccaaggatctgacccgcgcacaccgcgtggcacgcg
aaatcgaagcaggcatggtgtgggtgaactcctccaacgattccgatttccgcatcccattcggcggcgtgaag
cagtccggcatcggccgcgaactgggcgaagcaggcctggcaccatactgcaacgtgaagtccatccacgtgaa
cctggcagcataa

>TMABADH.2

atgtcctccaacgtgttcgtggaactgaagaccccagtgaccggcacctacaagcagccaaccggcctgttcat
aaacaacgaattcgtggaaggcgtggataagaagaccttcgaagtgatcaacccagcaaccgaagaagtgatct
gctccgtgcacgaagcaaccgaaaaggatgtggatatcgcagtggcagcagcacgcaaggcattcgaaggcgtg
tggcgcgatgtgaccccacagcagcgcggcatctacctgctgaagctggcagatctgctggaaaagaacctgga
tctgctggcagcagtggaatccctggataacggcaagtccatcaccatggcacgcggcgatgtgggcgcagtgg
tgggcaccatccgctactacggcggctgggcagataagatcgaaggcaagaccatcgatatctccccagattcc
ttccactacacccgccaggaaccactgggcgtgtgcggccagatcatcccatggaacttcccactgctgatgct
ggcatggaaggtgggcccagcactggcaaccggcaacaccatcgtgatgaagaccgcagaacagaccccactgt
ccgcactggtgttcgcacagttcgtgaaggaagcaggcttcccaccaggcgtgctgaacatcatctccggcttc
ggccgcatcgcaggcgcagcaatggcatcccacatggatatcgataaggtggcattcaccggctccaccatggt
gggccgccagatcatgaaggcagcagcagaatccaacctgaagaaggtgaccctggaactgggcggcaagtccc
caaacatcatcttcaacgatgcagatatcgatcaggcaatcgattgggtgaacttcggcatctacttcaaccac
ggccagacctgctgcgcaggctcccgcgtatacgtgcaggaaggcatctacgataagttcgtggcagcattcaa
gcagcgcgcacagcagaacaaggtgggcgatccattccacgatgaaaccttccagggcccacaggtgtcccagc
tgcagtacgatcgcatcatgggctacatcaaggcaggcaaggaagaaggcgcaaccgtggaaaccggcggcgaa
cgccacggcgataagggctacttcatccagccaaccatcttcaccaacgtgcgccacgatatgaagatcatgaa
ggaagaaatcttcggcccagtgtgcgcagtggcaaagttctccaccgaagaagaagtgatcaagctgggcaacg
attccaactacggcctggcagcagcagtgcacaccaaggatctgaacaccgcaatccgcgtgtccaaccacctg
cgcgcaggcaccgtgtgggtgaacacctacaacgcactgcaccaccagctgccattcggcggctacaaggaatc
cggcatcggccgcgaactgggcgaagcagcactggcaaactacacccagtgcaagtccgtggcaatcaagctga
actaa

6 Synthesis of TMABA

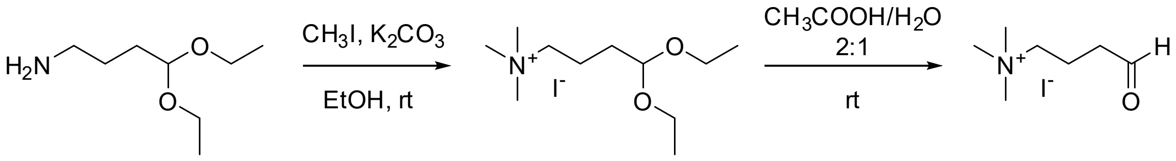

1 **2** **3**

Iodomethane (637 µl, 10.2 mmol, 3.3 eq.) was added to a solution of 4-aminobutyraldehyde diethyl acetal (**1**, 540 µl, 3.1 mmol, 1 eq.) and potassium carbonate (600 mg, 4.3 mmol, 1.4 eq) in Ethanol (6.2 mL). After stirring for 66 h at room temperature (rt), the reaction mixture was filtered and the filtrate was concentrated in vacuum. The remainder was suspended in warm diethylether, filtered and the solid was dried under reduced pressure to quantitatively yield 4-trimethylaminobutyraldehyde diethyl acetal iodide (**2**) as a colorless solid. For the deprotection of the aldehyde, the intermediate **2** (500 mg, 1.5 mmol) was dissolved in a mixture of acetic acid/water (2:1, 15 mL) and stirred for 4 h at rt. The solvent was removed under vacuum to yield 4-trimethylaminobutyraldehyde (TMABA) as an iodide salt in form of a yellow solid.

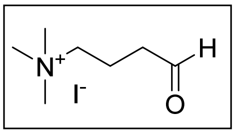

Yield: quant. ^{1}H NMR (500 MHz, DMSO-d_6): δ [ppm] = 9.68 (t, ^3J = 0.9 Hz, 1H, C**H**O), 3.26 (m, 2H, C**H₂**N(CH₃)₃), 3.06 (s, 9H, N(C**H₃**)₃), 2.57 (td, ^3J = 7.0, 0.9 Hz, 2H, C**H₂**CHO), 1.92 (m, 2H, C**H₂**CH₂CHO). $C_7H_{16}NOI$ (257.115 g mol^{-1})
MS(ESI): *m/z* = 130.1 (130.1 [M]$^+$)

7 References

Amann, E., Ochs, B., and Abel, K. J. (1988). Tightly regulated tac promoter vectors useful for the expression of unfused and fused proteins in *Escherichia coli*. *Gene* 69, 301–315. doi:10.1016/0378-1119(88)90440-4.

Baba, T., Ara, T., Hasegawa, M., Takai, Y., Okumura, Y., Baba, M., et al. (2006). Construction of *Escherichia coli* K-12 in-frame, single-gene knockout mutants: the Keio collection. *Mol. Syst. Biol.* 2, 2006.0008. doi:10.1038/msb4100050.

Datsenko, K. A., and Wanner, B. L. (2000). One-step inactivation of chromosomal genes in *Escherichia coli* K-12 using PCR products. *Proc. Natl. Acad. Sci. U.S.A.* 97, 6640–6645. doi:10.1073/pnas.120163297.

Franken, J., Burger, A., Swiegers, J. H., and Bauer, F. F. (2015). Reconstruction of the carnitine biosynthesis pathway from *Neurospora crassa* in the yeast *Saccharomyces cerevisiae*. *Appl. Microbiol. Biotechnol.* 99, 6377–6389. doi:10.1007/s00253-015-6561-x.

Gibson, D. G., Young, L., Chuang, R.-Y., Venter, J. C., Hutchison, C. A., and Smith, H. O. (2009). Enzymatic assembly of DNA molecules up to several hundred kilobases. *Nat. Methods* 6, 343–345. doi:10.1038/nmeth.1318.

Hanahan, D. (1983). Studies on transformation of *Escherichia coli* with plasmids. *J. Mol. Biol.* 166, 557–580. doi:10.1016/s0022-2836(83)80284-8.

Kang, W.-K., Le, B.-U. D. of B. S., Park, Y.-H. 705-102 M. L. A., Koh, E.-S. 101-1802 W. M. A., Ju, J.-Y. 104-401 M. D. A., Lee, J.-H. 104-1504 H. A., et al. (2013). A microorganism of enterobacteriaceae genus haboring genes associated with L-carnitine biosynthesis and method of producing L-carnitine using the microorganism. Available at: https://patents.google.com/patent/EP1904620B1/en [Accessed September 8, 2020].

Kirchner, O., and Tauch, A. (2003). Tools for genetic engineering in the amino acid-producing bacterium Corynebacterium glutamicum. *J. Biotechnol.* 104, 287–299. doi:10.1016/s0168-1656(03)00148-2.

Kugler, P., Fröhlich, D., and Wendisch, V. F. (2020). Development of a Biosensor for Crotonobetaine-CoA Ligase Screening Based on the Elucidation of *Escherichia coli* Carnitine Metabolism. *ACS Synth. Biol.* 9, 2460–2471. doi:10.1021/acssynbio.0c00234.

Swiegers, J. H., Vaz, F. M., Pretorius, I. S., Wanders, R. J. A., and Bauer, F. F. (2002). Carnitine biosynthesis in *Neurospora crassa*: identification of a cDNA coding for ε-*N*-trimethyllysine hydroxylase and its functional expression in *Saccharomyces cerevisiae*. *FEMS Microbiol. Lett.* 210, 19–23. doi:10.1111/j.1574-6968.2002.tb11154.x.
